# EHMTI-0217. Neurophysiological study of tdcs effects in healthy volunteers

**DOI:** 10.1186/1129-2377-15-S1-E3

**Published:** 2014-09-18

**Authors:** R Baschi, E Vecchio, SL Sava, V De Pasqua, J Schoenen, D Magis

**Affiliations:** 1 Department of Neurology, CHR Citadelle, Liege, Belgium; 2 Department of Neuroscience and Sense Organs, University of Bari, Bari, Italy

## Introduction

Transcranial direct current stimulation (tDCS) is a non-invasive neuromodulation technique able to activate (anode) or to inhibit (cathode) the underlying cortex. It could be of interest for the preventive treatment of migraine that is associated with interictal changes in cortical responsivity. To optimize neuromodulation protocols, however, studies of their physiological effects on the normal human brain are necessary.

## Aims

To study short and long-term effects of tDCS with the novel tDCS Cefaly° device on visual cortex and DLPFC in healthy volunteers (HV).

## Methods

Nine HV received anodal tDCS over Oz, 9 others anodalF3tDCS with the cathode at Oz: intensity 2 mA, duration 20min. We recorded CHEPS, QST, nBR and VEP at baseline (T0), immediately after tDCS (T1) and after 5 daily stimulations (T5).

## Results

Anodal tDCS over Oz increased VEP habituation at T1 and even more so at T5 (p=0.038). It also increased heat pain thresholds (p=0.02) and CHEPS habituation at T5 (p=0.035), while reducing its latency (p=0.04).The F3 anode–Oz cathode combination only reduced the nBR sensory threshold at T1 (p=0.039).

## Conclusions

Activation the visual cortex with anodal tDCS increases habituation of cortical evoked potentials and heat pain thresholds. This stimulation protocol may thus be of interest in migraine prevention. It also confirms our previous finding of an inhibitory connection between visual cortex and pain processing pathways.

No conflict of interest.

